# Laser Shock Fabrication of Nitrogen Doped Inverse Spinel Fe_3_O_4_/Carbon Nanosheet Film Electrodes towards Hydrogen Evolution Reactions in Alkaline Media

**DOI:** 10.3390/ijms23137477

**Published:** 2022-07-05

**Authors:** Dun Wu, Jiaming Zhao, Junfeng Cheng, Chunlin Liu, Qiang Wang

**Affiliations:** 1 Jiangsu Key Laboratory of Environmentally Friendly Polymeric Materials, School of Materials Science and Engineering, Changzhou University, Changzhou 213164, China; wudun@cczu.edu.cn (D.W.); 20085600106@smail.cczu.edu.cn (J.Z.); junfeng@cczu.edu.cn (J.C.); chunlin@cczu.edu.cn (C.L.); 2 Mechanical and Material Engineering Department, Changzhou University Huaide College, Jingjiang 214500, China

**Keywords:** laser ablation, photothermal agent, hydrogen evolution reaction

## Abstract

The reliable and cost-effective production of high-performance film electrodes for hydrogen evolution reactions remains a challenge for the laser surface modification community. In this study, prior to a thermal imidization reaction, a small number of Fe_3_O_4_ nanoparticles were vortexed into a poly(amic acid) (PAA) prepolymer, and the achieved flat composite film was then ablated by a 1064 nm fiber laser. After laser irradiation, the hierarchical architectures of carbon nanosheets decorated with Fe_3_O_4_ nanoparticles were generated. Although pure polyimide (PI) film and laser carbonized PI film, as well as bare Fe_3_O_4_, showcase poor intrinsic catalytic activity toward alkaline hydrogen evolution reactions, our laser-derived Fe_3_O_4_/carbon nanosheet hybrid film demonstrated enhanced electrocatalytic activity and stability in 1 M KOH electrolyte; the overpotential(η_10_) reached 247 mV when the current density was 10 mA cm^−2^ with a slight current decay in the chronoamperometric examination of 12 h. Finally, we proposed that the substitution of N to O in Fe−O sites of trans spinel structured magnetite would be able to modulate the free energy of hydrogen adsorption (ΔG_H*_) and accelerate water dissociation.

## 1. Introduction

The emerging challenge to make cheap and green hydrogen instead of hydrogen from fossil fuels is an essential prerequisite for achieving carbon neutrality by 2050 or 2060 [1]. Due to the intermittent merits of renewable power and generation capacities of present electricity grids, in situ energy production by water electrolysis and the distribution of hydrogen gas will enable the complete utilization of renewable energy [2]. As for the cathodic half reaction of the electricity-driven water-splitting hydrogen evolution reaction (HER), Ni-based catalysts and noble metal-based catalysts (Pt and RuO_2_) were generally deployed for the commercial mature alkaline water electrolyzer and the developing proton exchange membrane (PEM) water electrolyzer, respectively [3]. To look for an alternative to precious metals and develop active and durable electrocatalysts able to boost the hydrogen evolution reaction process, a variety of transition metal alloys and compounds and novel design strategies, such as defect, hetero-junction, strain, and d-band engineering, among others, have been evaluated comprehensively [4,5].

Since the discovery of laser-induced graphene (LIG) in 2014, increasing research efforts have been elicited to refine this protocol. For example, we reported a polyhedral carbon film through the laser scribing of a polyimide (PI) film with a 1064 nm laser [6,7]. To address the issue that PI is almost transparent at this laser wavelength and to improve the electrodes’ conductivity, we suggest the application of the typical photothermal therapy (PTT) agents in tumor theranostics that are usually triggered by near-infrared (NIR-Ⅱ) light (1000–1700 nm), and the use of magnetite (Fe_3_O_4_) nanoparticles exhibiting higher heating efficiency than hypothermia treatment initiated by alternating electromagnetic fields. As a narrow band-gap semiconductor (~0.3 eV), anti-spinel Fe_3_O_4_ possesses Fe (II) and half of Fe (III) ions in the octahedral sites and results in an inter-valence charge transfer that arises to a strong absorption in the NIR-Ⅱ region [8,9,10].

Although remarkable performance has been demonstrated and delineated in theory using transition-metal spinel oxides as electrocatalysts in oxygen-reduction reactions (ORR) and oxygen-evolution reactions (OER), the original trans spinel Fe_3_O_4_ has been seldom reported as an electrocatalyst for hydrogen evolution reactions due to the formation of strong hydrogen bonds between adsorbed H* intermediates and lattice O atoms within Fe_3_O_4_, as well as the relatively strong bond strength between iron and adsorbed hydrogen [11,12]. Furthermore, the feedstock of PI film and laser-ablated PI film were found to be inactive towards hydrogen evolution reactions in alkaline electrolytes in spite of the rich topological defects discovered in LIG [13]. This work creates a new platform used for the first time to convert magnetite, an earth-abundant mineral, into the robust HER electrocatalyst by encapsulating it into a polymer matrix and through scribing it by laser in one minute.

## 2. Results and Discussion

**Figure 1:**
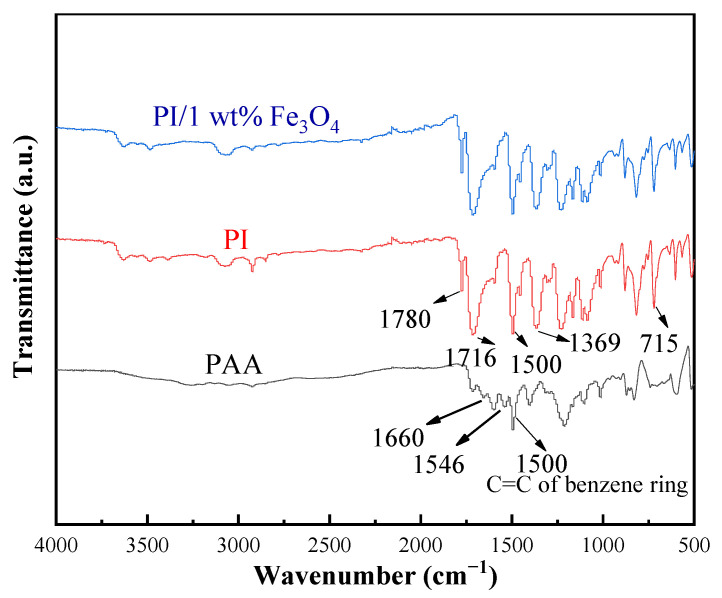
Fourier transform infrared spectra of PAA, PI, and Fe_3_O_4_/PI nanocomposite films.

Fourier transform infrared spectroscopy (FTIR) was carried out, as shown in Figure 1. It is well known that PI films are usually synthesized from poly(amic acid) (PAA) by thermal imidization at a high temperature. Dehydration with the cyclization step makes the polar groups of -COOH in PAA turn into imide rings in PI molecular. In the black line in Figure 1, the characteristic absorption bands at about 1660 cm^−1^ and 1546 cm^−1^ can be assigned to the carbonyl stretching vibration and N-H variable angle vibration, as well as the C-N stretching vibration of the amide in the PAA unit. The characteristic peaks at wavenumbers of 1780, 1716, 1369, and 715 cm^−1^ are mainly attributed to the characteristic vibrational modes of the imide rings of the PI unit in the red line in Figure 1. At a temperature of 350 °C, the characteristic absorption bands from the PAA unit disappear completely, indicating the conversion from PAA to PI.

Furthermore, the multiple relative ratio of the areas of FTIR absorption peaks at 1369 cm^−1^ (C-N stretching of the imide ring) and 1500 cm^−1^ (C=C stretching of the benzene block) is a descriptor of the proportion of the converted PAA, and the ratio of $\frac{S1369/S1500\;\left(\mathrm{black}\;\mathrm{line}\right)}{S1369/S1500\;\left(\mathrm{red}\;\mathrm{line}\right)}$ equals 1.22 (>1) in our case, which confirms the entire conversion of PAA [14]. From the blue line of Figure 1, it was found the corresponding spectra of the Fe_3_O_4_/PI composite film were the same as the pure PI system, which suggests the addition of a small amount of nanofiller of Fe_3_O_4_ into polymers did not change the thermal imidization process of the PAA.

**Figure 2:**
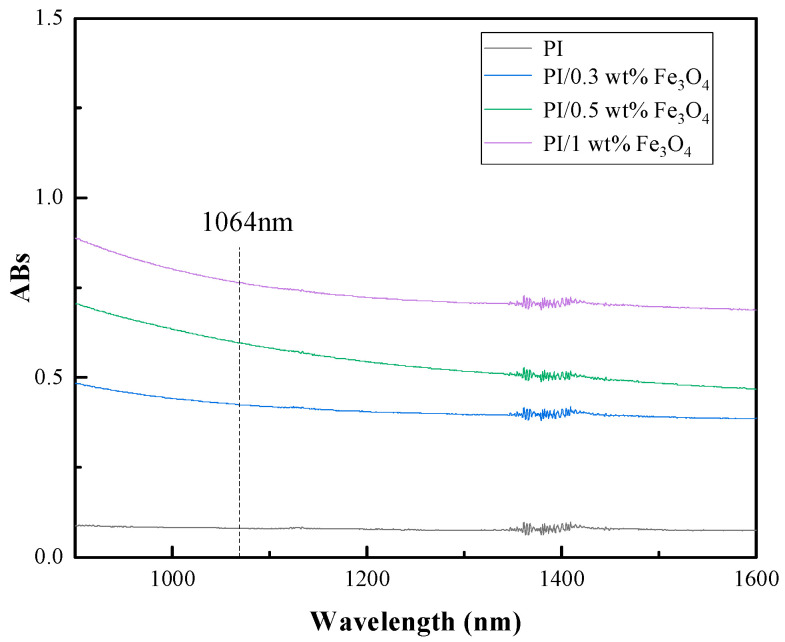
Near-infrared light absorption pattern of Fe_3_O_4_/PI nanocomposite film.

The absorption properties of PI composite films containing different concentrations of Fe_3_O_4_ towards near-infrared light (NIR-Ⅱ) are revealed in Figure 2. The PI film containing 0 wt% Fe_3_O_4_ nanoparticles displayed very weak absorption abilities in this light region. It was obvious that as the content of Fe_3_O_4_ increased (0.3–1 wt%), the absorption value of the composite film dramatically rose in the near-infrared region. In particular, the absorption value of the PI film with 1 wt% Fe_3_O_4_ nanoparticles was more than 0.8 for 1064 nm light, indicating that a small amount of Fe_3_O_4_ nanoparticles can effectively adsorb the incident laser light and generate more heat. Due to the difficulties of homogeneous dispersion and the alignment of hybrid film, a higher content of Fe_3_O_4_ nanoparticle composite film was not to be optimized at present.

**Figure 3:**
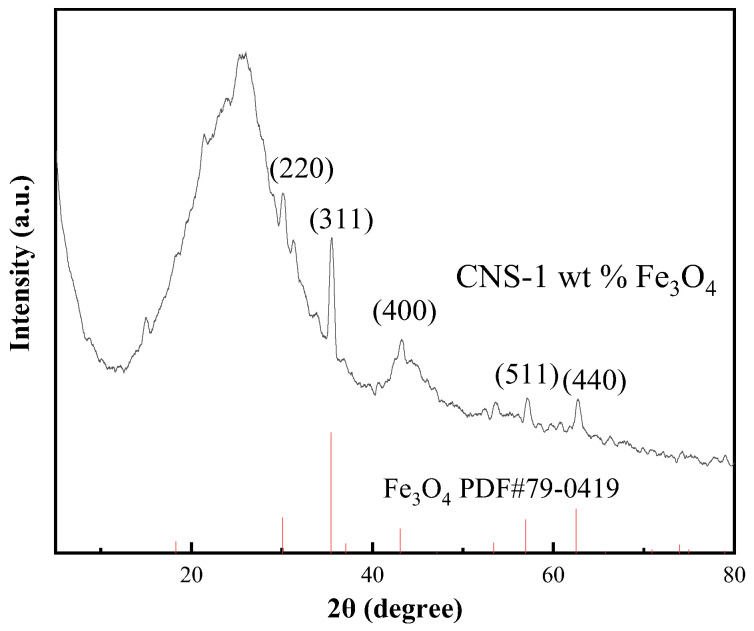
XRD patterns of the scratched powder after laser ablation.

To identify the composition and crystalline phase of the hybrid film (CNS-1 wt% Fe_3_O_4_) after laser ablation, the XRD patterns of the powder directly scratched from the film were analyzed, as shown in Figure 3. The broadened Bragg peaks at 26° and 43° are part of the characteristic diffraction pattern of turbostatic carbon nanostructures; a disorder stacked lattice planes along the (002) direction of graphitic carbon [15,16]. Another set of sharp diffraction peaks can be indexed to cubic trans spinel structured Fe_3_O_4_ (PDF 79-0419). Neither metallic iron nor iron carbides were detected in the samples after the laser ablation, in consideration of the possible carbothermal reduction reaction at the extreme high temperature and the instantaneous plasma states induced by laser beams. In other words, Fe_3_O_4_ nanopowder filled in the carbon matrix was stable under the irradiation of a 1064 nm laser at a high power.

**Figure 4:**
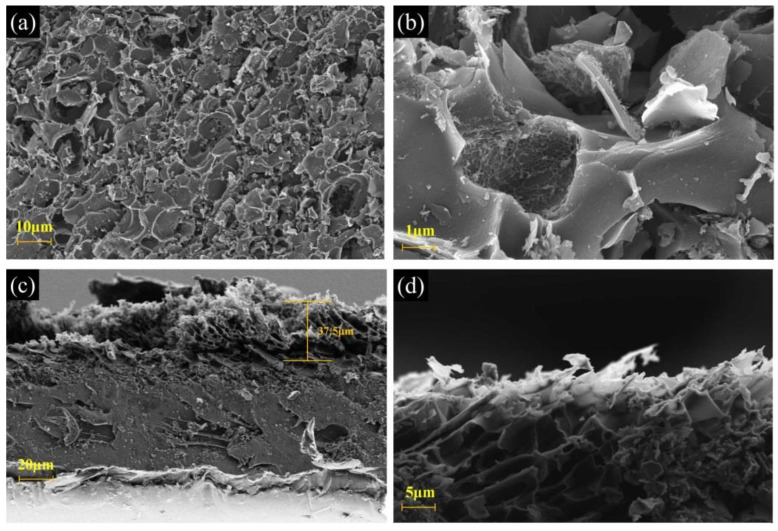
(**a**,**b**) Top view SEM of 1 wt% Fe_3_O_4_/CNS film, (**c**) sectional view of SEM of 1 wt% Fe_3_O_4_/CNS film, (**d**) high magnification SEM image of the film.

The microstructures of the as-prepared Fe_3_O_4_/CNS nanocomposite films were investigated by scanning electron microscopy (SEM) and transmission electron microscopy (TEM). Figure 4a,b is the top view of a scanning electron microscope image. Unlike pristine smooth Fe_3_O_4_/PI film, a 3D porous morphology was formed on the topmost layer of the composite film after the laser ablation, which might have been caused by the release of discomposed volatile gas during the laser ablation. The periodic folded nanoflakes with a thickness of less than 100 nm are more easily seen, as depicted in Figure S1. According to the cross-sectional scanning electron microscope image of the abated composite film in Figure 4c, the total thickness of the carbonized layer is about 37.5 μm. The high magnification image in Figure 4d shows that these carbon nanosheets directly grow on the substrate and can be integrated into a self-supporting film. More importantly, this unique alignment of carbon nanosheets could be beneficial for alleviating the aggregation of gas bubbles and facilitating the detachment of H_2_ from electrodes in water electrolysis. Finally, it should be noted that the backside of the ablated film was believed to be gently carbonized due to the visible color variation [17].

**Figure 5:**
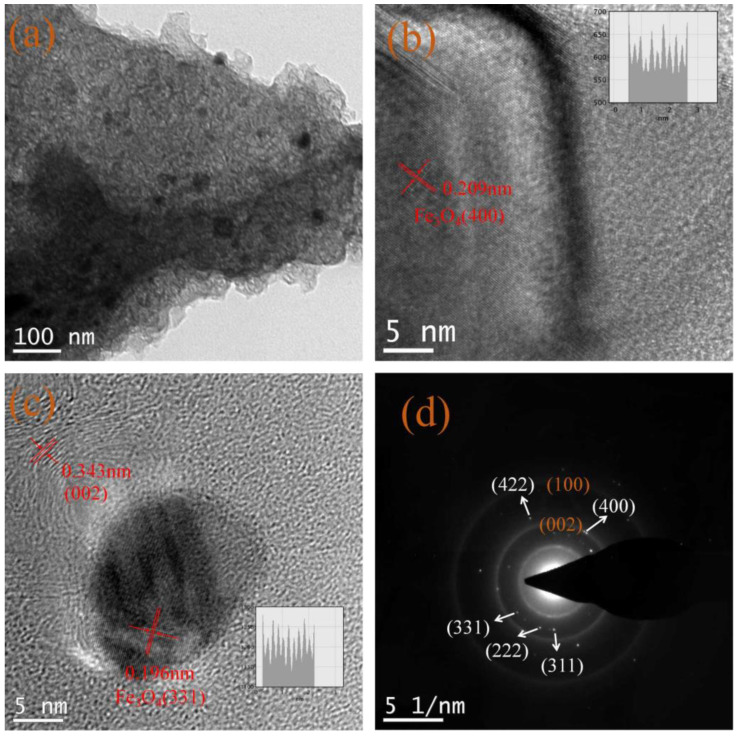
(**a**) The typical TEM image of CNS-supported Fe_3_O_4_ particles, (**b**,**c**) high magnification TEM (HRTEM) of the corresponding Fe_3_O_4_ nanoparticles; inset is the diagram of lattice fringes offered by the DM software, (**d**) the representative SAED pattern of the hybrid powder.

The transmission electron microscope image in Figure 5a reveals that 30 nm super-paramagnetic Fe_3_O_4_ nanoparticles are loaded homogenously on carbon nanosheets and no obvious agglomeration of Fe_3_O_4_ can be found. In the high-resolution TEM images of Figure 5b,c, the lattice fringes of Fe_3_O_4_ are about 0.209 nm and 0.196 nm, and can be designated to the (400) and (331) crystalline planes of Fe_3_O_4_, respectively. Furthermore, Figure 5c also exhibits a curved multi-layer graphene structure (~12 layers) with clear lattice fringes with spacing of a distance of 0.343 nm. The typical selected area of the electron diffraction pattern of Figure 5d presents (002) and (100) diffraction rings of carbon materials, as well as sharp diffraction spots of single-crystal Nano-Fe_3_O_4_ in its nature.

**Figure 6:**
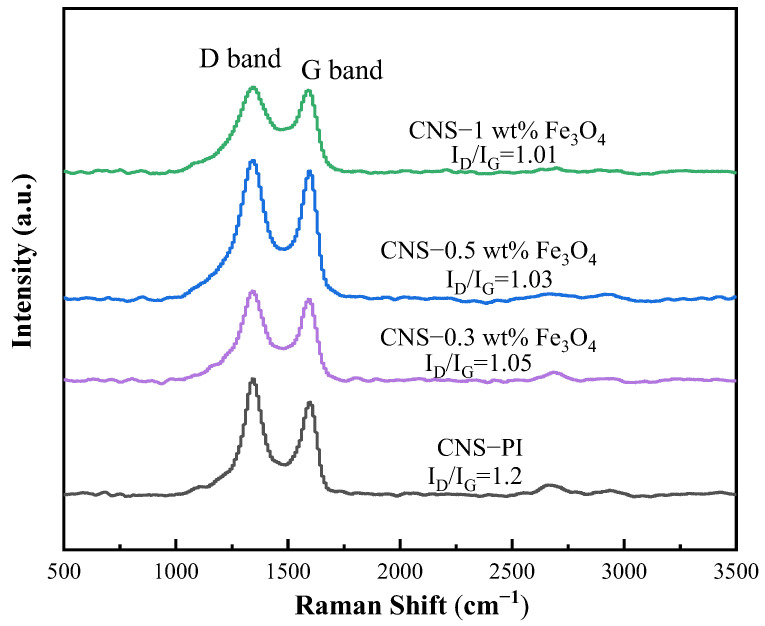
Raman spectra of laser-ablated Fe_3_O_4_/PI composite films with different Fe_3_O_4_ content.

Raman spectroscopy was employed to analyze the subtle structures of the carbon matrix. As described in Figure 6, three Raman peaks at about 1347 cm^−1^, 1598 cm^−1^, and 2680 cm^−1^ are designated as D, G, and 2D bands of the graphite structure, respectively [18]. As an index of the graphitic degree of the carbon materials, the relative intensity ratio of the D to G-band (I_D_/I_G_) showed a decreased trend in the order of the content of Fe_3_O_4_ in PI films, definitely indicating that the Fe_3_O_4_ species could effectively promote the transfer of energy from laser to heat and facilitate the degree of graphitization for carbon matrixes. A highly graphitized carbon matrix is not only favorable for improving electrode conductivity, but helps to promote electrode stability at industrial temperatures (50–80 °C).

**Figure 7:**
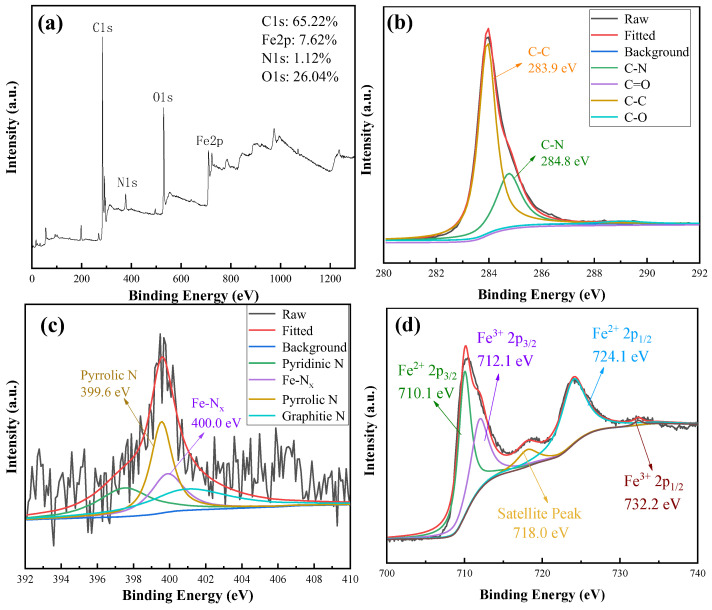
XPS spectra of 1 wt% Fe_3_O_4_/CNS composite film: (**a**) XPS survey of the samples, (**b**) the high-resolution C1s XPS spectra, (**c**) the high-resolution N1s XPS spectra, (**d**) the high-resolution Fe2p XPS spectra.

X-ray photoelectron spectroscopy (XPS) measurements were carried out to achieve insight into the elemental composition and atomic configurations of the laser-ablated films. Figure 7a presents XPS survey spectra of the samples, and the signals for C1s, N1s, O1s, and Fe2p peaks can be identified as having a content of 65.22 atom%, 1.12 atom %, 26.04 atom %, and 7.62 atom %, respectively. As displayed in Figure 7b, the high-resolution C1s spectra could be assigned to four peaks, corresponding to C-C, C-O, C=O, and C-N bonds, respectively. Combining polar groups with a carbon substrate is capable of regulating the hydrophilicity of carbon electrodes. The high-resolution N1s spectra of Figure 7c reveal the dominant pyrrolic N (399.6 eV) and Fe-N_x_ (400.0 eV) atom sites in total N atoms. The presence of an Fe-N_x_ moiety could be due to the fact that some of the lattice O atoms of the inverse spinel Fe_3_O_4_ are substituted by N atoms under the harsh laser irradiation. In particular, this Fe-N_x_ bond among the carbon matrix was regarded as being the efficient active site for the HER process [19]. In the high-resolution Fe2p spectrum of Figure 7d, no zero-valence metallic Fe^0^ was observed at 707 eV (719.9 eV). The pair of peaks at 710.3 eV and 724.1 eV should be assigned to Fe^2+^ 2p_3/2_ (2p_1/2_) of Fe_3_O_4_ and another pair of peaks of 712.1 eV and 732.2 eV could be ascribed to Fe^3+^ 2p_3/2_ (2p_1/2_) of the magnetite. In addition, the broadening satellite peak of Fe2p at 718.0 eV could be identified [20].

**Figure 8:**
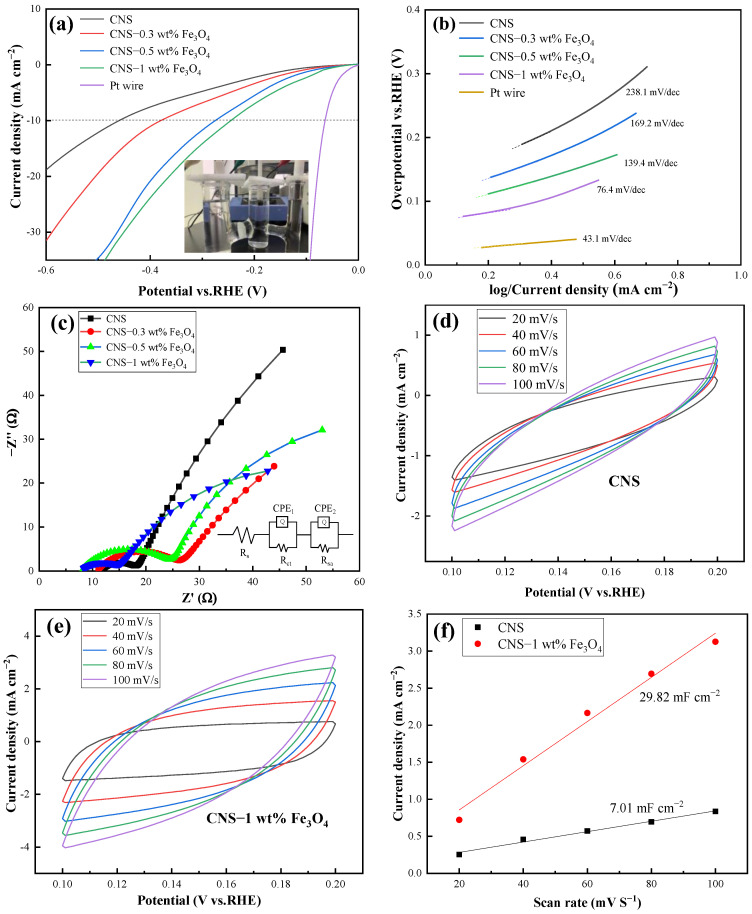
Electrochemical measurements in 1 M KOH. (**a**) LSV curves (inset is the photograph of the experimental setup), (**b**) Tafel curves, (**c**) Nyquist plots, (**d**,**e**) CV curve at different scan rates, (**f**) double-layer capacitances (Cdl).

The low-cost and alkaline-efficient electrocatalysts were expected to be of vast importance due to present alkaline water electrolysis being at an industry scale. Thus, linear sweep voltammetry (LSV) was first conducted at a scan rate of 2 mV s^−1^ in 1.0 M KOH aqueous electrolyte. As depicted in Figure 8a, to afford the current density of 10 mA cm^−2^ (the current density expected for 12.3% efficient solar water splitting), the ablated pure PI film demonstrated a higher overpotential of 456 mV that was in accordance with the results of Tour’s group (>700 mV) by employing another IR laser beam, suggesting the poor hydrogen evolving activity of the as-prepared PI film [21]. Moreover, bare Fe_3_O_4_ is seldom used as an HER catalyst due to the formation of strong hydrogen bonds with Had intermediates; thus, it becomes poisonous easily. Nevertheless, as the content of Fe_3_O_4_ rises from 0 wt% to 1 wt% in the laser scanned Fe_3_O_4_/PI film, the required overpotential of the ablated films finally decreases to 247 mV. Although it is still inferior to the benchmark of metallic platinum filament (63 mV in the same medium), this result is far superior to that of the Ni film catalyst (322 mV at 10 mA cm^–2^) and unambiguously reveals the feasibility of producing higher HER activity electrodes from inert precursors by laser irradiation [22]. To elucidate the reaction kinetics, Tafel plots derived from the LSV curves were performed (Figure 8b). The electrode of 1 wt% Fe_3_O_4_/CNS possesses a far lower Tafel slope of 76.4 mV dec^−1^ in comparison with the counterparts larger than 130 mV dec^−1^, indicating the catalytic kinetics of an HER should be guided by the Volmer–Heyrovsky mechanistic pathway with the Heyrovsky step determined as the rate-determining step [23]. To obtain deeper insight into the HER catalytic activity of the above electrode, we examined the electrode kinetics using electrochemical impedance spectroscopy (EIS). Nyquist plots displayed in Figure 8c demonstrated that the 1 wt% Fe_3_O_4_/CNS electrode possessed the smallest semicircular diameter in the high-frequency region (related to the charge transfer resistance Rct), confirming fast electron transportation in the catalysis process. Moreover, the data in the low-frequency region suggested that the electrode response was more complicated than a solely diffusion-controlled response.

Given the finite geometric area of electrodes, a higher electrochemical surface area (ECSA) usually denotes more active sites exposed to electrolytes. Figure 8f showed the double-layer capacitance (Cdl) (in proportion to ECSA) of 1 wt% Fe_3_O_4_/CNS and CNS electrodes, which are achieved via cyclic voltammetry (CV) measurements as a function of scan rates (Figure 8d,e) within a non-faradic reaction region. Remarkably, the calculated Cdl of the 1 wt% Fe_3_O_4_/CNS electrode is 29.82 mF cm^−2^, far higher than the 7.01 mF cm^−2^ of the CNS electrode, suggesting its higher number of potential active sites among catalysts.

**Figure 9:**
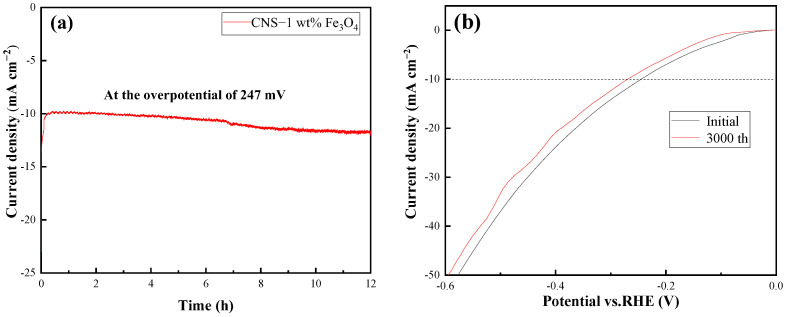
(**a**) Chronoamperometric responses at the overpotential of 247 mV, (**b**) HER polarization curves of the Fe_3_O_4_/CNS electrode before and after 3000 CV.

Apart from intrinsic catalysis activities, long-term stability is another crucial issue to evaluate for advanced electrocatalysts. To assess the stability of the 1 wt% Fe_3_O_4_/CNS catalysts, an accelerated degradation study was recorded in 1.0 M KOH. The chronoamperometric examination exhibited in Figure 9a indicated that the HER current density for our electrode almost remained unchanged at the overpotential of 247 mV for 12 h. This result was also confirmed by the polarization plots after the 1st and 3000th continuous cyclic voltammograms (CVs), the overpotential at 10 mA cm^−1^ slightly shifting from 247 mV to 270 mV, as illustrated in Figure 9b.

In the pursuit of unraveling the real catalytic configurations towards the HER of the Fe_3_O_4_/CNS catalysts, two possible water molecule dissociation mechanisms were discussed on the ground of the reported possible catalytic sites. In theory, the ΔG_H*_ for an ideal HER catalyst should be close to zero, which is favorable for H* adsorption and desorption, thus facilitating the proton–electron transfer process [24]. From this point of view, pure Fe_3_O_4_ is not an ideal HER catalytic candidate due to its high affinity to the intermediate H* and water molecule, leading to a relatively lower value of ΔG_H*_ [25]. In spite of the presence of intrinsic structure defects, as well as the Lewis basic carbon atom bonding to the adjacent N atom to break the electroneutrality of graphite and induce catalytic activity among the laser-treated CNS, the present experimental data support the notion that there might be a lack of high concentration of active sites exposed to accelerate the HER rate in the alkaline medium [26]. Regarding the extreme laser ablation circumstances, atom moieties, such as Fe-N_4_, Fe-N_3_O, or single-atom iron, might be created in the carbon matrix. As an important platinum-group metal-free (PGM-free) electrocatalyst, the well-established planar Fe-N_4_ model has provided kinetic insight into its excellent HER features. However, this assumption requires more than two N atoms to chelate with one Fe atom and obviously mismatches with our XPS survey results of a low ratio of N/Fe (0.15) [27,28]. Therefore, heteroatom-doped Fe_3_O_4_ should be a reasonable component for uncovering the impressive HER performance of our Fe_3_O_4_/CNS catalysts. Table 1 lists the critical parameters of typical alkaline HER electrocatalysts of iron oxides doped with different anions in 1M KOH [29,30,31,32,33].

**Figure 10:**
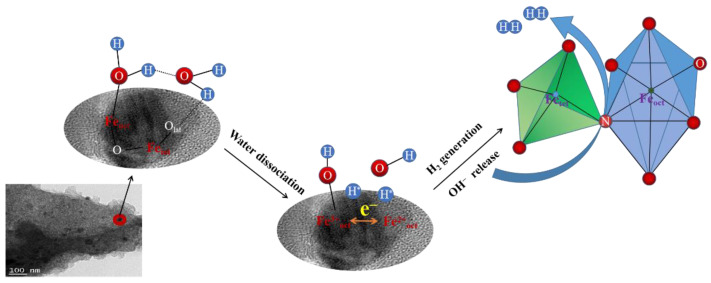
Schematic description of a possible reaction pathway of water dimer dissociation on the surface of N-Fe_3_O_4_ catalysts to disclose enhanced HER activity in alkaline media.

To simulate the realistic aqueous electrolyte condition and to overcome the high kinetic energy barriers for water dissociation above the surface of Fe_3_O_4_, an interesting bimolecular Volmer reaction pathway was established on the basis of the advancement of operando spectroscopic techniques; that is, water molecules are usually adsorbed on the surface of the catalysts in the form of dimer. Specifically, the single O atom of a water molecule is trapped on the Fe_oct_ site, while the H in another water molecule links with the O_lattice_ of Fe_3_O_4_ via a hydrogen bond, and the two water molecules are connected through H-bonding, creating a unique eight-membered ring [25], as described in Figure 10. By the substitution of O with N atoms in the Fe_3_O_4_ lattice, which was verified in our XPS analysis, the ΔG_H*_ of Fe_tet_ and Fe_oct_ is modulated to move in the direction of a zero energy level and optimizes the electron configuration of the inert original O atoms through interaction with the *p*- orbital electrons of N and P atoms, thus preventing the poisonousness of inverse spinel Fe_3_O_4_. Moreover, the delocalized electrons generated from the shifts between the Fe(II) and Fe(III) species in octahedral sites are helpful to the dissociated hydroxyl groups for capturing electrons to be OH^−^ anions from the N-Fe_3_O_4_ domains [33].

## 3. Materials and Methods

### 3.1. Fabrication of Fe_3_O_4_/CNS Nanocomposite Films

**Figure 11:**
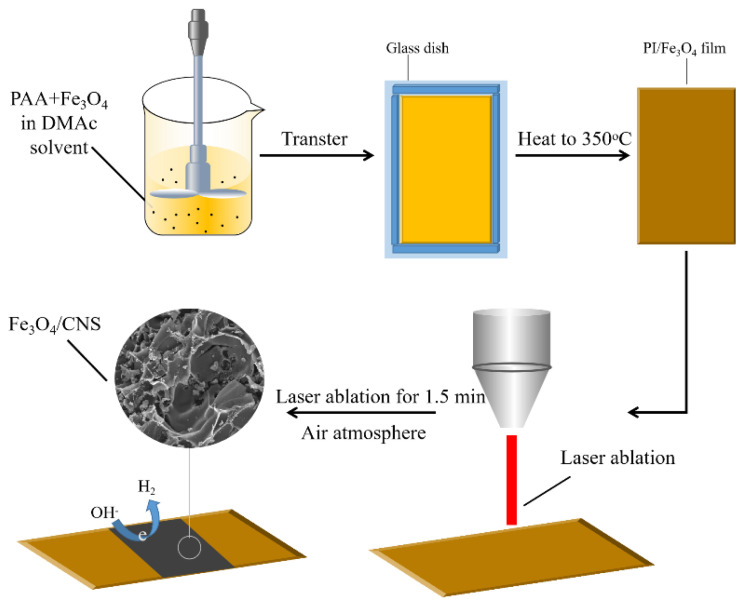
Schematic diagram of the fabrication process of the Fe_3_O_4_/CNS nanocomposite film electrodes by laser shock.

The fabrication process of the Fe_3_O_4_/CNS nanocomposite film electrodes by laser shock is illustrated in Figure 11.

Different amounts of Fe_3_O_4_ (~45 mg) at a size of ~30 nm (Figure S2, Aladdin Chemical, Shanghai, China) were ultrasound dispersed in 30 mL N,N-Dimethylacetamide (DMAC, Sinopharm Chemical Reagent Co. Ltd., Shanghai, China), which was then poured into 30 g PAA prepolymer (Suzhou Yuxin Tiancai New Material Application Technology Co., Ltd., Suzhou, China) with 15% solid content. After being fully stirred with a mechanical agitator, the colloidal dispersion was coated on the custom-made glass molds and transferred to the vacuum pumping to remove the air bubbles with the programmable heating at 350 °C. After the thermo curing, dense and flat Fe_3_O_4_/PI composite film was obtained by pressing it under a flat vulcanizing machine (YF-8017, Yangzhou Yuanfeng Experimental Machine Factory, Yangzhou, China, Figure S3) at 350 °C. Laser ablation of Fe_3_O_4_/PI film was conducted in a smart fiber laser marking machine (FMF20W, Changzhou Xinfang Industrial Intelligent Equipment Co., Ltd., Changzhou, China). With the aim of obtaining hybrid film electrodes with good electrical conductivity, we adjusted the parameters of the laser machine. The optimum values are a scanning speed of 220 mm/s, line spacing of 0.001 mm, pulse frequency of 30 KHz, and power of 2.4 W. The relations of laser power and the square resistances of the hybrid film are recorded in Figure S4. All the laser experiments proceeded with the raster mode in the air under ambient conditions, as shown in Figure S5 and Movie S1.

### 3.2. Materials Characterization

The thermal imidization reaction was monitored with the Fourier Transform Infrared Spectrometer (Nicolet iS10, Thermo Fisher Scientific, Waltham, MA, USA). UV-VIS-NIR diffuse reflectance spectrophotometry (UV-3600, Shimadzu, Kyoto, Japan) was performed to investigate the absorption values of Fe_3_O_4_/PI composite film in the near-infrared region. The X-ray powder diffraction (XRD) patterns of the samples were collected using a D/max 2500 PC (Rigaku, Kyoto, Japan) diffractometer with monochromatic Cu Ka radiation at a wavelength of 0.1541 nm and at a scanning speed of 2° min^−1^. The morphologies of the samples were observed by a field emission scanning electron microscope, FE-SEM (Zeiss, Jena, Germany, supra55), with acceleration voltage of 5 kV at Secondary Electron mode; TEM and high-resolution transmission electron microscopy (HR-TEM) were taken using a Tecnai G2 F30 (FEI) transmission electron microscope with acceleration voltage of 200 kV at Bright Field mode; Raman spectra were recorded on a confocal Thermo Fisher DXR Raman microscope with the wavelength of the incident laser at 532 nm and the power at 7 mW.; X-ray photoelectron spectra (XPS) were obtained on a VG ESCA Lab MK II X-ray photoelectron spectrometer with an exciting source of Al- Ka (hν = 1486.6 eV). In the XPS spectra, all binding energies were referenced to the C 1s neutral carbon peak at 284.5 eV and the elemental compositions were determined from peak area ratios calculated with software.

### 3.3. Electrochemical Tests

The deionized water (DI water, R = 18.2MΩ) used in all experiments was purified with a Millipore system. The electrochemical measurements were carried out in a typical three-electrode system with three-cell setup using an electrochemical work station (CHI660E, Shanghai Chenhua Instrument Co., Ltd., Shanghai, China) in N_2_-saturated 1.0 M KOH solution (pH = 13.6) at 25 °C. Ag/AgCl electrode (3.5 M KCl solution), and a graphite rod was employed for the reference and counter electrodes. The Fe_3_O_4_/carbon nanosheet film after laser ablation was tailored directly as the working electrode with a fixed area of (5 mm × 5 mm), and the activity of the catalyst was normalized by square areas. All of the potentials were converted to the reversible hydrogen electrode (RHE) according to the equation E (vs RHE) = E (vs Ag/AgCl) + 0.197 V + 0.059 × pH. The scanning rate of the polarization curve for the HER was set at 2 mV s^–1^. All of the LSV tests were automatically corrected using current interrupt iR compensation. Chronopotentiometric measurements were conducted to evaluate long-term stability. The determination of ECSA was calculated by measuring the CV curves at different scan rates (20, 40, 60, 80, and 100 mV s^–1^). EIS was performed in constant potential mode at open circuit potential over a frequency range from 100 kHz to 0.1 Hz.

## 4. Conclusions

In summary, through the incorporation of a photothermal agent of nano-magnetite into the precursor of polyimide, a flat and dense Fe_3_O_4_-PI hybrid film can be achieved after thermal curing and showcases excellent near-infrared light absorption features. Being scanned with a 1064 nm laser beam, it can be transformed to composite electrodes of carbon nanosheets decorated with nitrogen-doped Fe_3_O_4_ nanoparticles. Due to the regulation of electronic configurations of Fe-O sites in anti-spinel Fe_3_O_4_ with doped N atoms, the composite electrode affords an impressive overpotential of 247 mV at the current density of 10 mA cm^−2^ in 1M KOH. The interesting assistance of the octahedral Fe sites of Fe_3_O_4_ is supposed to facilitate water dissociation through bi-molecule Volmer reaction pathways. Our developed fabricating strategy not only offers new insights into the facile synthesis of cheap Fe-based electrocatalysts towards hydrogen production, but can be extended for use in making advanced anodes for lithium-ion batteries, the electrodes of supercapacitors, and so on.

## Figures and Tables

**Table 1:** Comparison of the HER performance of similar catalysts in 1 M KOH.

Electrocatalysts	Over Potential (mV) j = 10 mA cm^−2^	Tafel Slope (mV dec^−1^)
LIG [6]	>700	None
CNS	456	238.1
Fe_3_O_4_-N/CNF [29]	300	119
Fe_2_O_3_-NCs800 [30]	245	76.6
S-Fe_3_O_4_/NF [31]	219	149.4
P-Fe_3_O_4_/IF [32]	138 (j = 100 mA cm^−2^)	41.9 (j = 100 mA cm^−2^)
CNS-1 wt% Fe_3_O_4_, this work	247	76.4
Pt wire	63	43.1

## Data Availability

Not applicable.

## Supplementary Materials

The following supporting information can be downloaded at: https://www.mdpi.com/article/10.3390/ijms23137477/s1.
