# Supplementary material for: Laser Shock Fabrication of Nitrogen Doped Inverse Spinel Fe3O4/Carbon Nanosheet Film Electrodes towards Hydrogen Evolution Reactions in Alkaline Media
Source: Int J Mol Sci. 2022 Jul 5;23(13):7477. doi: 10.3390/ijms23137477 (PMC9267510; doi:10.3390/ijms23137477)
Supplement: Supplementary file 1 [file ijms-23-07477-s001.zip › ijms-1793750-supplementary.pdf]

Supporting information

Laser Shock Fabrication of Nitrogen Doped Inverse Spinel Fe_3O_4/Carbon Nanosheet Film Electrodes towards Hydrogen Evolution Reactions in Alkaline Media

Dun Wu, Jiaming Zhao, Junfeng Cheng, Chunlin Liu and Qiang Wang *

Jiangsu Key Laboratory of Environmentally Friendly Polymeric Materials, School of Materials Science and Engineering, Changzhou University, Changzhou 213164, China; wudun@cczu.edu.cn (D.W.); 20085600106@smail.cczu.edu.cn (J.Z.); junfeng@cczu.edu.cn (J.C.); chunlin@cczu.edu.cn (C.L.)

* Correspondence: wq@cczu.edu.cn, Tel.: +86-519-86330100

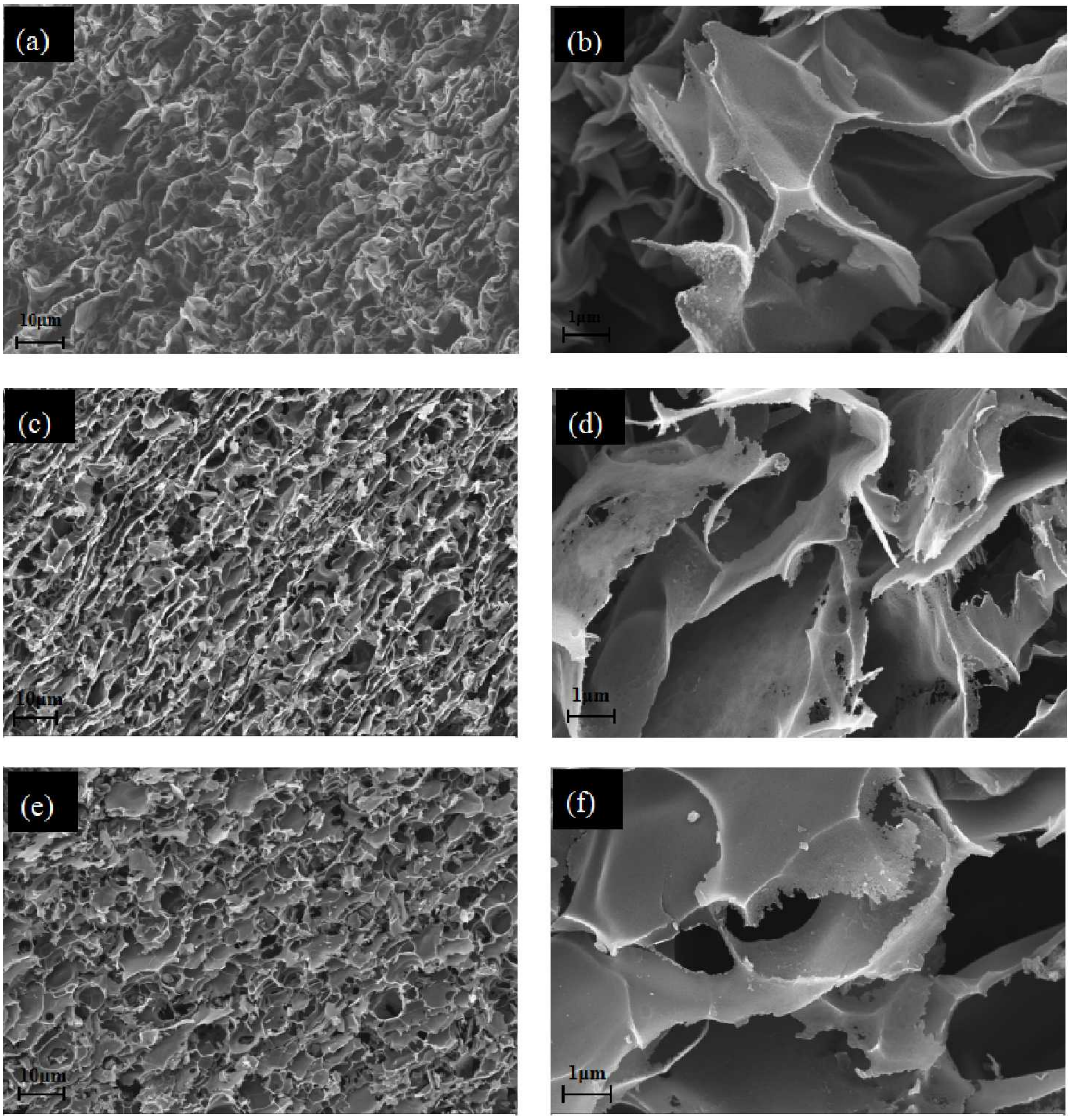

Figure S1. SEM of PI/Fe_3O_4 composite film ablated by laser: (a)(b) 0 wt% Fe_3O_4, (c)(d) 0.3 wt% Fe_3O_4, (e)(f) 0.5 wt% Fe_3O_4

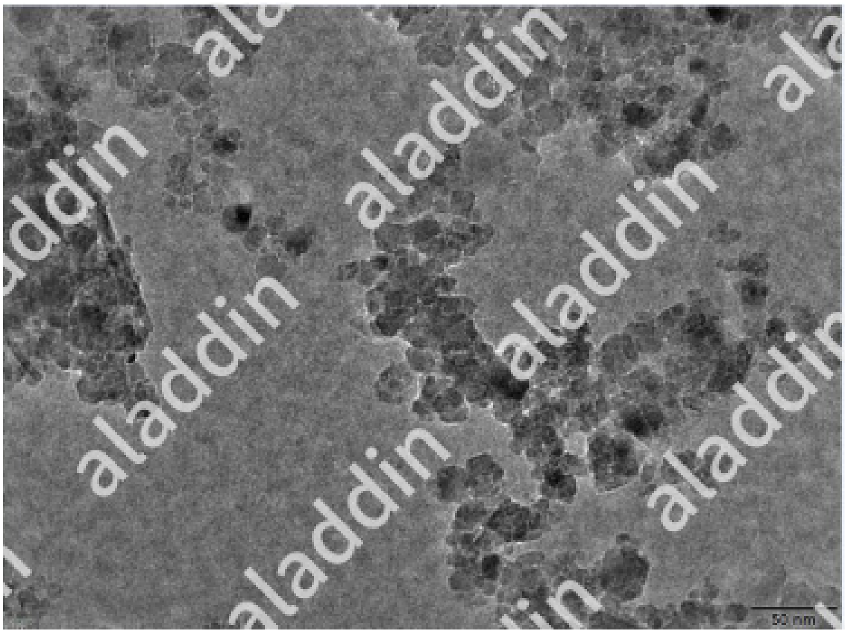

Figure S2. The typical TEM image of Nano-Fe_3O_4.

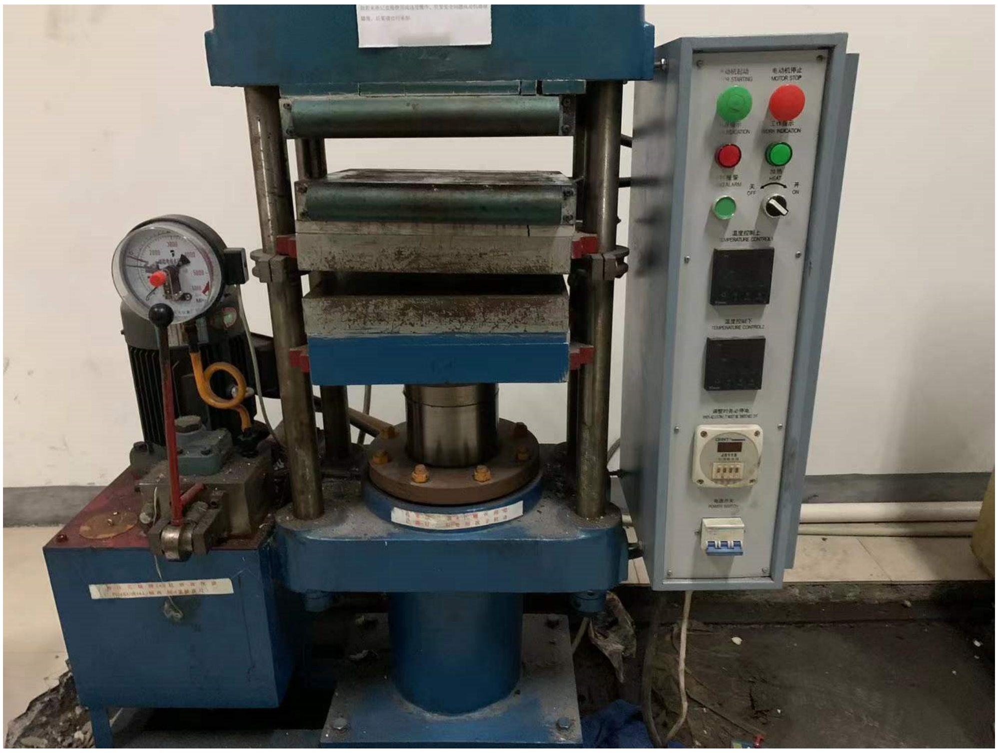

Figure S3. Flat vulcanizing machine.

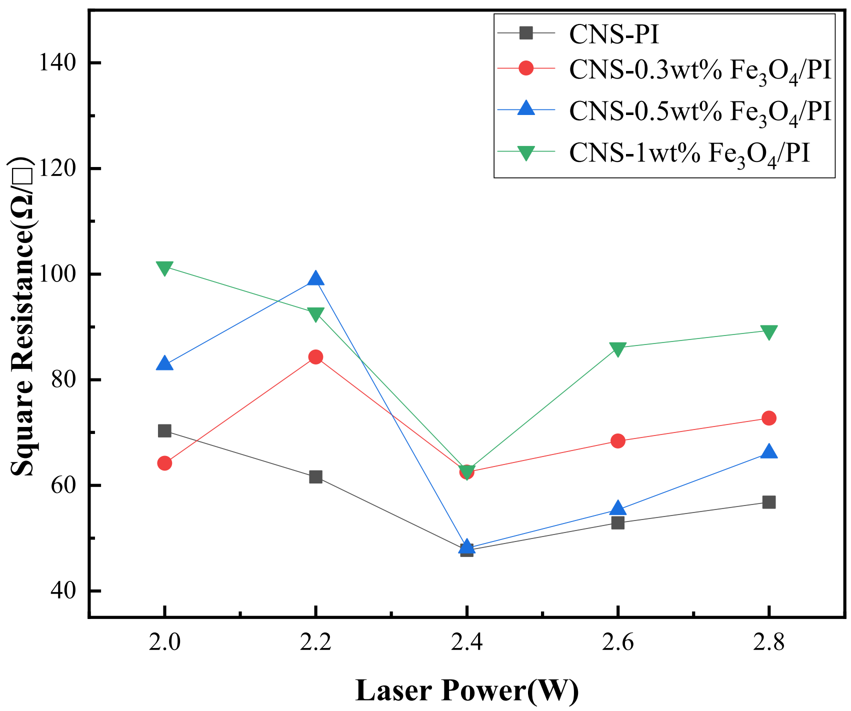

Figure S4. Square resistance of CNS-PI/Fe_3O_4 under different laser power.

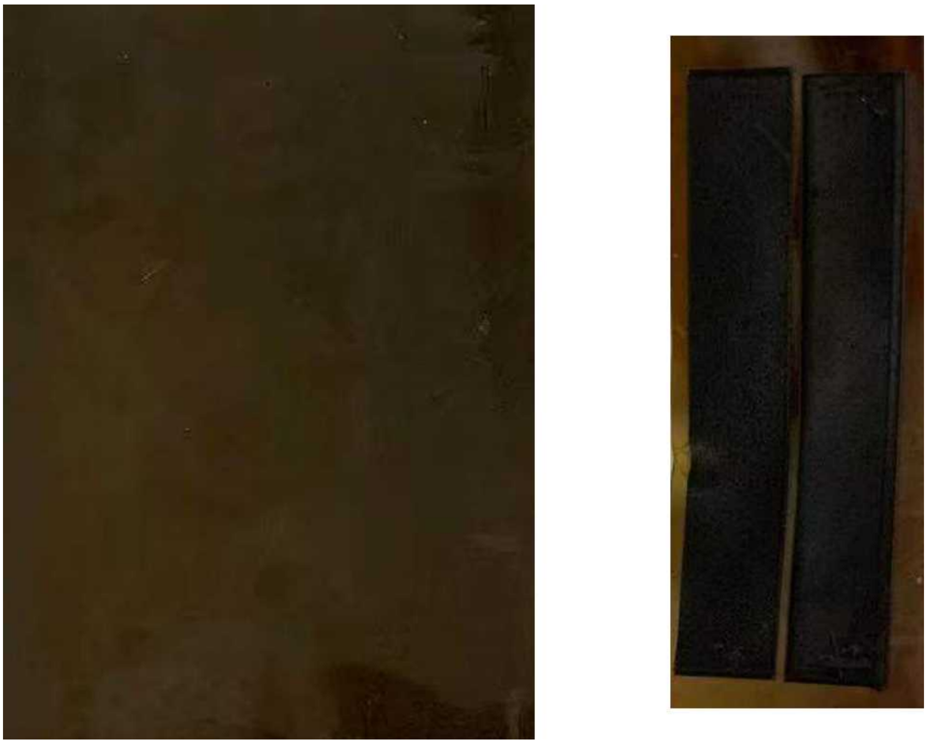

Figure S5. The photograph of Fe_3O_4/PI composite film before and after laser scribing.

Movie S1. Laser Ablate Video

061fbd8f0f5a5a0c9feba0eb276fef8f.mp4
